# Symptom burden in end-stage liver disease: a prospective cohort study of the symptoms experienced by patients and the role of palliative care

**DOI:** 10.1177/17562848251353624

**Published:** 2025-07-13

**Authors:** Hugo M. Oliveira, Filipa Ribeiro, Graça Lopes, Eliana Frias, Filipe Andrade, Verónica Guiomar, Eduardo Eiras, Francisca Rego, Rui Nunes

**Affiliations:** Department of Social Sciences and Health, Faculty of Medicine, University of Porto, Porto, Portugal; Palliative Care Unit, Matosinhos Local Health Unit, Hospital Pedro Hispano, Rua Dr. Eduardo Torres, Senhora da Hora, 4464-513 Matosinhos, Portugal; Palliative Care Unit, Matosinhos Local Health Unit, Matosinhos, Portugal; Palliative Care Unit, Matosinhos Local Health Unit, Matosinhos, Portugal; Palliative Care Unit, Matosinhos Local Health Unit, Matosinhos, Portugal; Hepato-Bilio-Pancreatic Oncology Group, Matosinhos Local Health Unit, Matosinhos, Portugal; Hepato-Bilio-Pancreatic Oncology Group, Matosinhos Local Health Unit, Matosinhos, Portugal; Hepato-Bilio-Pancreatic Oncology Group, Matosinhos Local Health Unit, Matosinhos, Portugal; Department of Social Sciences and Health, Faculty of Medicine, University of Porto, Porto, Portugal; Department of Social Sciences and Health, Faculty of Medicine, University of Porto, Porto, Portugal

**Keywords:** end-stage liver disease, hepatology, liver cirrhosis, pain, palliative care

## Abstract

**Background:**

Liver disease is a leading cause of morbidity and mortality. Patients with end-stage liver disease (ESLD) experience multiple physical symptoms. Despite the poor prognosis and significant symptom burden, palliative care integration remains limited.

**Objectives:**

To assess the symptom burden in ESLD patients, the viability of applying a symptom scale in routine evaluations, and to assess the impact of palliative care on symptom management.

**Design:**

Observational, prospective cohort study.

**Methods:**

We prospectively included patients with chronic liver disease following their first episode of decompensation or diagnosis of hepatocarcinoma (HCC). Data collected included patient demographics, ESLD etiology, history of decompensation, and patient-reported symptom burden. Two-sided tests were used to identify factors of disease severity and evaluate the benefits of palliative care intervention.

**Results:**

Forty-four patients were assessed, divided into two cohorts: palliative care cohort (52.3%; *n* = 23) and hepatology care cohort (47.7%; *n* = 21). Patients in the palliative care cohort were older (69.35 ± 11.71 vs 59.86 ± 7.11 years; *p* = 0.002), had lower functional status (59.13 ± 2.51 vs 72.38 ± 2.92; *p* = 0.002), and higher prevalence of unstable decompensated cirrhosis (60.9% vs 28.6%; *p* = 0.043) and HCC (*p* < 0.001). This cohort reported a higher overall symptom burden, with rates of 82.6% for asthenia, 65.2% for pain, and 56.5% for anorexia. Palliative care interventions tended to reduce the prevalence of pain, anorexia, and dyspnea, with a significant decrease in pain intensity from 86.7% to 23.1% (*p* = 0.008) and asthenia intensity from 100% to 84.2% (*p* < 0.001).

**Conclusion:**

Significant differences in symptom prevalence were observed between the two cohorts, likely due to specific clinical characteristics of each group. The use of a symptom assessment scale proved to be simple and effective, revealing a high prevalence of symptoms. Palliative care was associated with a positive impact on symptom management.

**Trial registration:**

NCT06181474.

## Plain language summary

Managing symptoms in advanced liver disease: how palliative care helps

Liver disease is a major health issue, with people with severe liver disease often facing many uncomfortable symptoms. This study aimed to understand the symptoms experienced by patients with severe liver disease, how these symptoms relate to the severity of their condition, and the way palliative care could help manage these symptoms. The researchers included patients who had recently experienced a worsening of their condition or were diagnosed with liver cancer. The study involved 44 patients, divided into two groups: one receiving palliative care (23 patients) and another receiving standard liver care (21 patients). The palliative care group had older patients with more severe symptoms. Palliative care was found to help reduce the pain and improve the management of other symptoms. The study found notable differences in symptoms between the two groups, likely because of the unique health conditions of each one of them. Overall, palliative care can have a positive effect on managing symptoms for those patients.

## Introduction

Liver cirrhosis represents an advanced stage in the continuum of liver disease, initially characterized by an asymptomatic period known as compensated cirrhosis. This phase can progress to decompensated cirrhosis when complications associated with portal hypertension or liver dysfunction, such as ascites, hepatic encephalopathy, or hepatocarcinoma (HCC), arise.^1,2^ The transition to the end-stage liver disease (ESLD) phase, through the appearance of one or more complications, translates into a reduction in survival, which, on average, is estimated at 2 years.^1^ In addition to these complications, some patients may develop acute-on-chronic liver failure (ACLF), a clinical syndrome characterized by multiple organ dysfunctions and high short-term mortality after an episode of liver decompensation.^3,4^ Recent evidence indicates that the clinical phenotype of each patient has a significant impact on prognosis.^5,6^ Patients may present with pre-ACLF, defined as acute decompensation of cirrhosis that progresses to ACLF within a 90-day follow-up period; unstable decompensated cirrhosis (UDC), characterized by rehospitalization due to acute decompensation within 3 months of the initial episode; or stable decompensated cirrhosis (SDC), referring to those who do not require rehospitalization for acute decompensation during the same timeframe.^5,6^

Liver disease is a leading global cause of morbidity and mortality, accounting for approximately 1–2 million deaths/year,^7,8^ primarily affecting the young adult population (25–64 years).^9^ The SUPPORT study indicates that patients experiencing their first hospitalization episode present a 29% mortality rate, with another 29% patients dying within the first year.^10^ This study demonstrated that patients with ESLD experience a marked decline in quality of life, characterized by a wide range of physical, cognitive, and social symptoms that are often challenging to manage.^10^ Commonly reported symptoms include pain, dyspnea, fatigue, and mental confusion.^10^ A 2019 meta-analysis conducted by Peng et al.^11^ reported symptom prevalence ranging from 30% to 79% for pain, 20% to 88% for dyspnea, 56% to 68% for muscle cramps, 52% to 86% for asthenia, and 47% to 64% for pruritus. Notably, the majority of the studies included in the analysis reported the presence of only one or two symptoms per patient.^11^

Palliative care is a specialized, multidisciplinary medical care that addresses the physical, psychological, social, and spiritual needs of patients with advanced illness and their caregivers, regardless of prognosis and survival.^12,13^ Furthermore, a pivotal study by Baumann et al.^14^ demonstrated that referral to palliative care for patients on the liver transplant list resulted in symptomatic improvement. Moreover, a growing body of evidence highlights the benefits of implementing palliative care in ESLD patients. In addition to symptomatic control, palliative care teams facilitate timely discussion of the care plan, helps in reducing unnecessary hospitalizations and in better-coordinated care, also being important in supporting caregivers,^10,15,16^ which led to publication by the American Association for the Study of the Liver of the first recommendations on integrating palliative care and symptom management in patients with decompensated cirrhosis and incorporating palliative care elements into multidisciplinary decision-making for patients with HCC.^17,18^ Despite increasing recognition of the benefits of early palliative care in ESLD, multiple studies have identified significant barriers to its implementation by specialists.^19,20^ These include the widespread misconception among patients that palliative care is exclusively associated with end of life, the continued prioritization of curative treatment strategies by attending physicians, and the insufficient number of available palliative care specialists.^19,20^

One crucial question that emerged is when to refer ESLD patients to palliative care. In fact, traditional models, such as the Supportive & Palliative Care Indicators Tool (SPICT) and the NECPAL CCOMS-ICO Tool^©^, identify patients with signs of deterioration in their health status, but fail to present objective data on the severity of underlying liver disease.^21,22^ Specific models for ESLD patients have been developed, aiming to demonstrate the relationship between several variables, like the Model for End-Stage Liver Disease (MELD) score, associated mortality, and the implementation of palliative care.^23–25^ However, these models, which tend to be mathematical, may not be able to demonstrate patient frailty or symptomatic burden. Effectively, studies show that even with low MELD scores, up to 70% of these patients may present with a high symptom burden.^26^

The primary objectives of this observational study are to assess the symptom burden experienced by ESLD patients, the viability of implementing a symptom scale in routine evaluations of ESLD patients, and to evaluate the intervention of palliative care on the symptom burden of ESLD patients. We also aim to explore the correlation of the symptom burden with MELD-sodium (MELD-Na) score and each patient’s clinical phenotype.

## Materials and methods

### Study design

**Figure 1.**
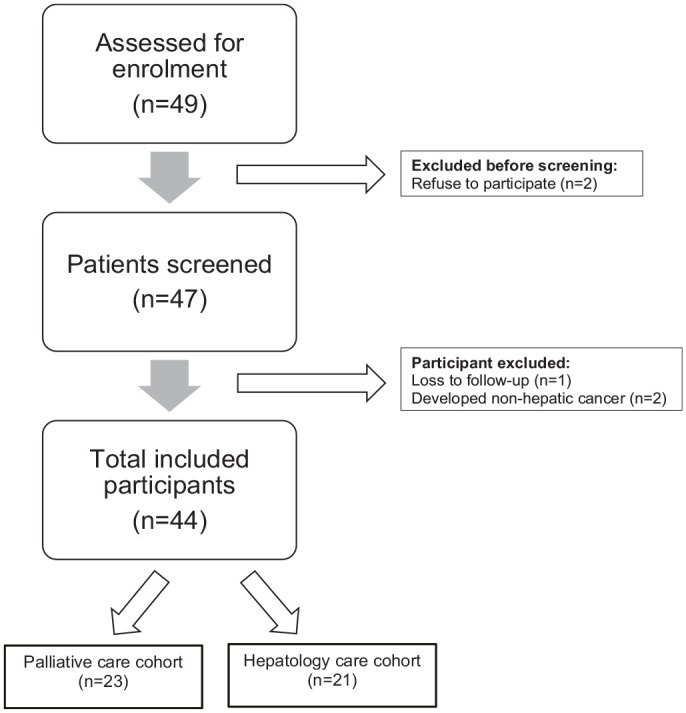
STROBE flow chart. STROBE, Strengthening the Reporting of Observational Studies in Epidemiology.

This study is an observational, prospective cohort study of patients with chronic liver disease following their first episode of decompensation or diagnosis of stage C or D HCC, according to the Barcelona Clinic Liver Cancer (BCLC) criteria.^27^ The reporting of this study conforms to the Strengthening the Reporting of Observational Studies in Epidemiology (STROBE) statement (Figure 1 and Supplemental Material).^28^

### Patients and setting

Between January and October 2024, all adult patients with chronic liver disease were consecutively enrolled following either their first episode of decompensation or a new diagnosis of HCC. Recruitment took place at a Portuguese secondary referral urban hospital equipped with comprehensive facilities, including integrated hepatology, oncology, and palliative care services. The study employed a convenience and purposive sampling strategy without randomization. Exclusion criteria included: (1) patients under 18 years old; (2) patients with chronic liver disease who died from causes unrelated to the disease progression; (3) presence of HCC without cirrhosis; (4) presence of non-hepatic cancer; and (5) persistent hepatic encephalopathy, psychiatric disorders, dementia, or other cognitive impairments preventing informed consent.

Eligible patients were approached by the study staff (which included a physician or a nurse) to obtain informed consent. Referrals to palliative care were carried out independently by the clinicians, without influence from the study staff. During the study period, a total of 49 patients were assessed for eligibility. Of these, five patients (10.2%) were excluded: two declined to participate, one was lost to follow-up, and two developed non-hepatic malignancies during the follow-up period. The final analysis included 44 patients, who were stratified into two cohorts: those referred to palliative care, comprising the palliative care cohort, and those managed exclusively by hepatology services, comprising the hepatology care cohort.

### Data collection

Clinical characteristics were collected from electronic medical records at the time of enrollment and during subsequent follow-up chart reviews. These included the etiology of liver disease, classified as alcohol-related, metabolic dysfunction-associated steatotic liver disease, viral hepatitis B or C, or other causes. Additional data comprised cirrhosis-related complications such as ascites, hepatic encephalopathy, and esophageal variceal bleeding; disease severity measured by the MELD-Na and Child-Pugh scores; functional status assessed using the Karnofsky Performance Scale; and comorbidities evaluated through the Charlson Comorbidity Index.^26,29–31^

Patient-reported symptom burden was assessed resorting to the revised Edmonton Symptom Assessment System (ESAS-r) scale.^32,33^ The ESAS-r evaluates the severity of nine common symptoms experienced by patients with serious illnesses (pain, tiredness, nausea, depression, anxiety, drowsiness, appetite, well-being, and shortness of breath), with an option to include a 10th symptom. We specifically included cramps and pruritus, which are prevalent in patients with chronic liver disease. Symptoms were rated on a numerical scale from 0 to 10 (0 indicating absence and 10 indicating the worst severity). The choice for this scale was due not only to its recurrent use among palliative care teams but also to existing studies demonstrating its applicability in patients with ESLD.^34^

All patients underwent a minimum of two assessments. In the palliative care cohort, symptoms were evaluated at the initial consultation and reassessed 5 days following the intervention. In the hepatology care cohort, assessments were conducted 1 month after the episode of decompensation and again 3 months later.

### Statistical analysis

Statistical analysis was carried out using Statistical Package for the Social Sciences for Mac (version 26), IBM^®^ SPSS^®^. Descriptive statistics were used to present continuous variables as mean and standard deviation, and categorical variables as frequency and percentage. For each item of the ESAS scale, we report the overall symptom prevalence and the percentage of patients with a moderate to severe symptom burden (defined as an individual item score ⩾4). The statistical significance of the differences between the two groups was assessed using the appropriate tests. Comparisons between categorical variables were performed with the Pearson’s χ^2^ test or Fisher’s exact test, where appropriate. Comparisons between continuous variables were carried out using Student’s *t* test for normally distributed variables. If it was not possible to apply the *t* test, we resorted to the nonparametric Mann-Whitney test. The nonparametric Wilcoxon test was used to compare related (paired) results. Statistical significance was determined by a two-sided *p*-value of less than 0.05, or a 95% confidence interval that did not include 1.

### Ethical considerations

The study protocol complied with the ethical principles outlined in the 1975 Declaration of Helsinki and was approved in advance by the institutional Ethics Committee (reference number 131/CES/JAS). Informed consent was obtained from all participants, who received no financial compensation for their participation. To ensure anonymity, all personal identifiers were removed, and each participant was assigned an alphanumeric code, accessible only to the principal investigator. The study was registered on ClinicalTrials.gov under the identification number NCT06181474.

## Results

### General characterization of the sample

A total of 44 patients were evaluated, and all their data were complete. They were divided into two cohorts: those referred to palliative care (52.3%; *n* = 23—palliative care cohort) and those receiving standard hepatology care (47.7%; *n* = 21—hepatology care cohort). Most patients were male, with similar distributions in both groups (Table 1). The average age of participants was 64.82 ± 10.80 years (Table 2), with the palliative care cohort being older compared to the hepatology care cohort (69.35 ± 11.71 vs 59.86 ± 7.11 years; *p* = 0.002). Karnofsky functional status revealed that the palliative care group had a lower average score compared to the hepatology cohort (59.13 ± 2.51 vs 72.38 ± 2.92; *p* = 0.002). According to the Charlson Comorbidity Index, patients in the palliative care cohort had a higher score versus those in the hepatology care cohort (6.52 ± 1.81 vs 3.95 ± 1.63; *p* < 0.001).

**Table 1.** Characterization of the population and underlying liver disease.

Characteristics of patients or disease	Palliative care cohort *n* (%)	Hepatology care cohort *n* (%)	Total *n* (%)	*p*
Gender				
Female	4 (17.4)	3 (14.3)	7 (15.9)	0.999
Male	19 (82.6)	18 (85.7)	37 (84.1)	
Chronic liver disease etiology				
Alcohol	15 (65.2)	17 (81.0)	32 (72.7)	0.242
Other	8 (34.8)	4 (19.0)	12 (27.3)	
Type of decompensation				
Ascites	9 (52.9)	8 (38.1)	17 (44.7)	n.d.
Hepatic encephalopathy	1 (5.9)	7 (33.3)	8 (21.1)	
Gastrointestinal bleeding	2 (11.8)	5 (23.8)	7 (18.4)	
Spontaneous bacterial peritonitis	1 (5.9)	1 (4.8)	2 (5.3)	
Hepatorenal syndrome	1 (5.9)	0 (0.0)	1 (2.6)	
ACLF	3 (17.6)	0 (0.0)	3 (7.9)	
Type of decompensation				
Ascites	9 (52.9)	8 (38.1)	17 (44.7)	0.360
Other	8 (47.1)	13 (61.9)	21 (55.3)	
Clinical phenotype				
Stable decompensated cirrhosis	7 (30.4)	14 (66.7)	21 (47.7)	**0.043**
Unstable decompensated cirrhosis	14 (60.9)	6 (28.6)	20 (45.5)	
Acute event	2 (8.7)	1 (4.8)	3 (6.8)	
Hepatocarcinoma				
Yes	17 (73.9)	0 (0.0)	17 (38.6)	**<0.001**
No	6 (26.1)	21 (100.0)	27 (61.4)	
Child-Pugh				
A	6 (26.1)	3 (14.3)	9 (20.5)	0.103
B	13 (56.5)	8 (38.1)	21 (47.7)	
C	4 (17.4)	10 (47.6)	14 (31.8)	
Death during follow-up				
Yes	20 (87.0)	2 (9.5)	22 (50.0)	**<0.001**
No	3 (13.0)	19 (90.5)	22 (50.0)	

*p*, significance level.

ACLF, acute-on-chronic liver failure; n.d., not detectable. Statistically significant findings are marked in bold.

**Table 2.** Characterization of age, Charlson index, Karnofsky score, MELD-Na, number of hospitalizations, and number of decompensations.

Variable	Total		Palliative care cohort		Hepatology care cohort		*p*
	*n* $\bar{X}$ ± *s*	IQR $\tilde{X}$	*n* $\bar{X}$ ± *s*	IQR $\tilde{X}$	*n* $\bar{X}$ ± *s*	IQR $\tilde{X}$	
Age	44 64.82 ± 10.80	57–74 63.00	23 69.35 ± 11.71	62–79.5 69.00	21 59.86 ± 7.11	56–63 59.00	**0.002**
Charlson index	44 5.30 ± 2.14	3–7 5–50	23 6.52 ± 1.81	6–8 7.00	21 3.95 ± 1.63	3–5 3.00	**0.000**
Karnofsky score	44 65.45 ± 14.22	50–80 80	23 59.13 ± 2.51	50.00–70.00 60.00	21 72.38 ± 2.92	60.0–80.0 70.0	**0.002**
MELD-Na	44 18.70 ± 6.66	15.0–22.0 19.0	23 20.43 ± 1.43	15.0–25.5 20.0	21 16.81 ± 1.31	13.0–20.0 18.0	0.071
Number of hospitalizations	44 1.52 ± 1.00	1–2 1.00	23 1.57 ± 0.95	1–2 2.00	21 1.48 ± 1.08	1–1 1.00	0.414
Number of decompensations	44 1.86 ± 1.25	1–3 1.00	23 2.09 ± 1.38	1–3 2.00	21 1.62 ± 1.07	1–2 1.00	0.219

$\bar{X}$ ± *s*, mean ± standard deviation; $\tilde{X}$ , median; *p*, significance level for comparing results between palliative care cohort and hepatology care cohort.

Statistically significant findings are marked in bold. IQR, interquartile range; MELD-Na, model for end-stage liver disease-sodium.

The main cause of ESLD was alcohol 72.7% (*n* = 32), with similar distributions in both groups. Main forms of decompensation included ascites (44.7%; *n* = 17) and hepatic encephalopathy (21.1%; *n* = 8), with similar distributions between cohorts (Table 1). The MELD-Na score documented in the population undergoing palliative care follow-up was 20.43 ± 1.43 versus 16.81 ± 1.31 (*p* = 0.071). Notably, there was a progressive improvement in the MELD-Na score over time for the hepatology cohort (from 16.81 ± 1.31 to 13.90 ± 1.15; *p* = 0.017).

Regarding the clinical phenotype, the palliative care group had a higher prevalence of UDC type (60.9% vs 28.6%; *p* = 0.043). Average hospitalization rates were similar for both cohorts (1.57 ± 0.95 vs 1.48 ± 1.08; *p* = 0.414), as were rates of decompensation (2.09 ± 1.38 vs 1.62 ± 1.07; *p* = 0.219). The prevalence of HCC in the study population was 38.6%, with 10 patients in BCLC stage C and 7 in stage D, all within the palliative care cohort (*p* < 0.001). During the follow-up period, 50% (*n* = 22) of the patients died, with 87% (*n* = 20) of those belonging to the palliative care cohort (*p* < 0.001).

### Symptomatic burden in ESLD patients

In the studied population, four symptoms were reported with a prevalence exceeding 30%: asthenia (77.3%), pain (43.2%), anorexia (43.2%), and drowsiness (34.1%). In contrast, the prevalence of cramps and pruritus (specific symptoms of ESLD) was low, at 9.1% and 0%, respectively.

**Figure 2.**
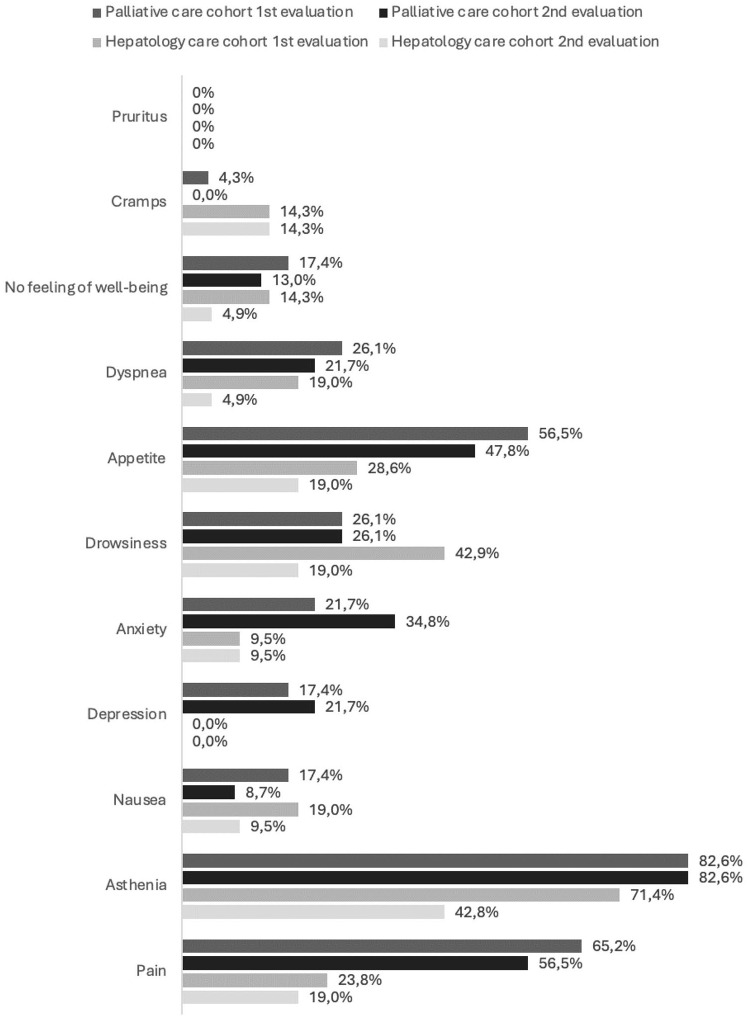
Prevalence of symptoms by moment of assessment in the populations under study.

The palliative care cohort exhibited (Figure 2) a higher overall symptom burden, with rates of 82.6% for asthenia, 65.2% for pain, and 56.5% for anorexia at initial assessment. Notably, all patients with asthenia reported a moderate to high intensity. In terms of pain, 86.7% (*n* = 13) rated it as moderate to high intensity.

In contrast, the hepatology care cohort exhibited a significantly higher proportion of asymptomatic patients and generally reported symptoms of lower intensity. At the initial assessment, the prevalence rates were 71.4% for asthenia, 42.9% for drowsiness, 28.6% for anorexia, and 23.8% for pain. Within this cohort, 46.7% (*n* = 7) of patients described asthenia as moderate to severe, while 44.4% (*n* = 4) rated drowsiness as of moderate intensity. By the 3-month reassessment following the initial decompensation event, the prevalence of asthenia and drowsiness had declined substantially to 42.8% and 19.0%, respectively.

### Impact of palliative care on symptom burden

On average, there were 44.43 ± 40.06 days between the initial decompensation and the evaluation by the palliative care team. Five days following the initial intervention by the palliative care team, the prevalence of pain decreased (Table 3) to 56.5%, anorexia to 47.8%, and dyspnea to 21.7%. Moderate to high intensity pain showed a reduction from 86.7% to 23.1% (*p* = 0.008). The prevalence of asthenia remained unchanged at 82.6%, but there was a reduction of moderate to high intensity asthenia, from 100% to 84.2% (*p* < 0.001). Furthermore, anxiety and depression symptoms worsened between the two assessments (Figure 2).

**Table 3.** Outcomes of symptoms by group and evaluation period.

Symptoms	Evaluation	Palliative care cohort		Hepatology care cohort		Fisher test^a^
		Symptoms		Symptoms		
		No	Yes	No	Yes	
Pain	First	34.78%	65.22%	76.19%	23.81%	**0.011**
	Second	43.48%	56.52%	80.95%	19.05%	**0.035**
McNemar test^b^		0.344		0.500		
Asthenia	First	17.39%	82.61%	28.57%	71.43%	**<0.001**
	Second	17.39%	82.61%	57.14%	42.86%	**0.007**
McNemar test^b^		0.750		**0.035**		
Nausea	First	82.61%	17.39%	80.95%	19.05%	0.422
	Second	91.30%	8.70%	90.48%	9.52%	0.999
McNemar test^b^		0.344		0.313		
Depression	First	82.61%	17.39%	100.00%	0.00%	0.240
	Second	78.26%	21.74%	100.00%	0.00%	0.075
McNemar test^b^		0.500		n.d.		
Anxiety	First	78.26%	21.74%	90.48%	9.52%	0.128
	Second	65.22%	34.78%	90.48%	9.52%	0.113
McNemar test^b^		0.188		0.999		
Drowsiness	First	73.91%	26.09%	57.14%	42.86%	0.420
	Second	73.91%	26.09%	80.95%	19.05%	0.059
Teste McNemar^b^		0.688		**0.031**		
Appetite	First	43.48%	56.52%	71.43%	28.57%	**0.009**
	Second	52.17%	47.83%	80.95%	19.05%	0.114
McNemar test^b^		0.313		0.250		
Dyspnea	First	73.91%	26.09%	80.95%	19.05%	0.744
	Second	78.26%	21.74%	95.24%	4.76%	0.354
McNemar test^b^		0.500		0.125		
No feeling of well-being	First	82.61%	17.39%	85.71%	14.29%	0.999
	Second	86.96%	13.04%	95.24%	4.76%	0.739
McNemar test^b^		0.500		0.250		
Cramps	First	95.65%	4.35%	85.71%	14.29%	0.100
	Second	100.00%	0.00%	85.71%	14.29%	0.100
McNemar test^b^		0.500		0.750		
Pruritus	First	100.00%	0.00%	100.00%	0.00%	n.d.
	Second	100.00%	0.00%	100.00%	0.00%	n.d.
McNemar test^b^		n.d.		n.d.		

a Fisher’s exact independence test was applied to infer the possible association of symptoms with the group under study at each assessment.

b McNemar test for each group considering the assessments performed (paired).

n.d., not detectable. Statistically significant findings are marked in bold.

During the follow-up period, the palliative care cohort exhibited a mortality rate of 87% (*n* = 20). The mean duration from the initial palliative care assessment to death was 55.55 ± 59.42 days, with 50% of patients surviving for at least 90 days following the assessment.

## Discussion

Our study shows the considerable symptom burden in patients with ESLD, with significant differences in symptom prevalence between the palliative care and hepatology care cohorts, possibly due to specific clinical characteristics of each group. We highlight that the palliative care group involved older patients, with a worse functional score and higher comorbidities. In this context, implementing a symptom assessment scale in routine evaluations proved to be simple and effective. Palliative care was associated with a reduction in symptom burden, spotlighting the need for its broader integration in ESLD care.

We documented a prevalence of asthenia, pain, anorexia, and drowsiness in this population, which closely aligns with the data reported by Peng et al.,^11^ corroborating findings from the SUPPORT study, which emphasizes the substantial symptom burden experienced by patients with ESLD.^10^ However, with no explanation found for this difference, the prevalence of cramps and pruritus in our study was significantly lower.^11^ We found considerable differences in symptom prevalence between patients referred to palliative care and those who were exclusively monitored by hepatology. Patients in the latter group exhibited a higher proportion of asymptomatic cases and generally reported symptoms of lower intensity.

Although the two cohorts shared similar characteristics, including the etiology of ESLD, type of decompensation, number of hospitalizations, and notably comparable MELD-Na scores, there may be no direct association between MELD score and symptomatic burden. This observation aligns with previous studies suggesting that the MELD scale does not reliably reflect either the need for palliative care or the intensity of symptomatology.^26,34–39^ We identified other specific differences between the populations. Patients in the palliative care cohort tended to be older, had lower functional status, and exhibited more comorbidities. These observations corroborate studies indicating that older patients are more likely to be referred to palliative care,^40–43^ in addition to support the classic models for identifying patients with deterioration in their health status, such as SPICT and NECPAL.^21,22^ In fact, the functional status appears to be the most consistent parameter between classical models and more specialized models for the ESLD population.^23–25^ In addition, some studies indicate that lower functional status is associated with a greater symptom burden,^44,45^ potentially explaining the higher symptom prevalence observed in the palliative care cohort. Although functional status may not be an ideal prognostic marker, it is a crucial indicator of frailty and palliative needs, reinforcing that referrals should be based on patient needs rather than on prognosis.

Our analysis revealed that the clinical phenotype of UDC was more prevalent in the palliative care cohort, which had a significantly higher mortality rate (87%). Conversely, the hepatology cohort frequently displayed the SDC phenotype, associated with a lower mortality rate. These findings reaffirm that patients with ESLD progressing to ACLF or UDC have a poorer prognosis, even in subclinical cases.^5,6,46^ It is not possible to establish a definitive association between clinical phenotype and symptom prevalence, as multiple interacting factors may influence this relationship. This notion is supported by findings from a study conducted by Hansen et al.,^39^ which had previously highlighted the complexity of these interactions. However, identifying these clinical phenotypes remains essential for optimizing the allocation of healthcare resources and ensuring more targeted and effective care strategies.

Furthermore, our study documented a high prevalence of HCC in the palliative care cohort, which demonstrates that referral to palliative care seems to be easier when ESLD patients are diagnosed with HCC,^37,40,43,47–49^ highlighting a potential direct association between cancer diagnosis and palliative care access.

The ESAS scale, which is adapted and validated in several languages, proved to be simple to implement, echoing findings from a recent study.^34^ There was a need for initial training in its application, but its regular practice allows to create a routine without increasing consultation time. We believe that the routine use of this scale could facilitate the prompt identification of symptoms requiring intervention in patients with ESLD. Nevertheless, future research should explore feasibility in different clinical settings.

We demonstrated that intervention by the palliative care team led to a reduction in the prevalence of pain, anorexia, and dyspnea, with a significant reduction in pain intensity. In addition, while the prevalence of asthenia remained unchanged, its intensity also decreased. The benefit of implementing palliative care in the symptomatic improvement of patients with ESLD has already been referred to in the pioneering study carried out by Baumann et al.^14^ However, published studies about this topic and the optimal pharmacological strategies for this population are limited.^50^ An increasing prevalence of anxiety and depression has also been observed, potentially linked to heightened awareness of disease progression. This underscores the critical importance of integrating psychological support within palliative care teams to address the profound emotional response many patients experience, helping them coping with ESLD.^51^

It is important to foreground that in the hepatology cohort, the stabilization and improvement of ESLD through medical management of ascites and hepatic encephalopathy, as well as nutritional support, contributed to symptom alleviation, strengthening what is mentioned in international recommendations about the importance of the best possible control of ESLD complications in symptom control.^17^

Moreover, there are also other data that should be spotlighted. The referral rate for palliative care in our study was 52.3%, which is consistent with recent literature and higher than previously reported data in our country,^35,48,52–54^ which indicates a growing integration of palliative care in ESLD management. We also observed a marked increase in the average time between palliative care assessment and death, with 50% of patients presenting a period of at least 90 days, representing a significant improvement over previous reports.^38,53^

There are some limitations to our study, notably the unicentric nature that limits data generalization, as well as the sample size, which was smaller than our initial goal, potentially reducing statistical power and limiting detection of other significant differences, namely in the analysis of palliative care on symptom management. Nevertheless, the sample reflects our national scale and is close to the study published by Shinall et al.^55^ We must also underline that the palliative care group patients had specific characteristics: they were older patients, had a worse functional score, and a high percentage of HCC, which may impact the symptoms described. The challenges in patient enrollment and follow-up required adjustments to our initial protocol, which aimed for quarterly evaluations or assessments following each decompensation episode in the hepatology cohort.

Despite these limitations, our study provides noteworthy insights: it applies a recognized symptomatic assessment scale to all ESLD patients, demonstrating a considerable prevalence of symptoms; it showed a growing a positive impact of palliative care on symptom management; and it emphasizes the critical role of the prompt identification of symptoms in promoting timely referrals to palliative care for ESLD patients. Furthermore, we can consider this as a pilot study with the aim of progressing toward a multicentric analysis.

## Conclusion

Patients with ESLD carry a significant symptom burden. The routine use of symptom assessment scales is effective in facilitating the timely identification of symptoms requiring intervention. The positive impact of palliative care on patients with ESLD highlights the importance of its broader integration into standard ESLD management.

## Supplemental Material

sj-docx-1-tag-10.1177_17562848251353624 – Supplemental material for Symptom burden in end-stage liver disease: a prospective cohort study of the symptoms experienced by patients and the role of palliative care Supplemental material, sj-docx-1-tag-10.1177_17562848251353624 for Symptom burden in end-stage liver disease: a prospective cohort study of the symptoms experienced by patients and the role of palliative care by Hugo M. Oliveira, Filipa Ribeiro, Graça Lopes, Eliana Frias, Filipe Andrade, Verónica Guiomar, Eduardo Eiras, Francisca Rego and Rui Nunes in Therapeutic Advances in Gastroenterology

## Appendix

### Abbreviations

ACLF acute-on-chronic liver failure

BCLC Barcelona Clinic Liver Cancer

ESAS-r Edmonton Symptom Assessment System revised

ESLD  end-stage liver disease

HCC hepatocarcinoma

MELD-Na model for end-stage liver disease-Na

SDC stable decompensated cirrhosis

SPICT supportive and palliative care indicators tool

UDC unstable decompensated cirrhosis

## Declarations

**Ethics approval and consent to participate:** The study protocol followed the ethical guidelines of the 1975 Declaration of Helsinki and was approved by the ethical commission of Matosinhos Local Health Unity (131/CES/JAS). Informed verbal and written consent was obtained from the patients for publication.

**Consent for publication:** All authors approve this manuscript, and the copyright is transferred to *Therapeutic Advances in Gastroenterology*.

**Availability of data and materials:** None.

## References

[1] D’Amico G Garcia-Tsao G Pagliaro L . Natural history and prognostic indicators of survival in cirrhosis: a systematic review of 118 studies. J Hepatol 2006; 44(1): 217–231. 16298014 10.1016/j.jhep.2005.10.013

[2] de Franchis R Bosch J Garcia-Tsao G , et al.; Baveno VII Faculty. Baveno VII—renewing consensus in portal hypertension (published correction appears in *J Hepatol* 2022; 77(1): 271. doi: 10.1016/j.jhep.2022.03.024). J Hepatol 2022; 76(4): 959–974. 35120736 10.1016/j.jhep.2021.12.022 PMC11090185

[3] Perricone G Jalan R . Acute-on-chronic liver failure: a distinct clinical syndrome that has reclassified cirrhosis. Clin Liver Dis (Hoboken) 2019; 14(5): 171–175. 31879558 10.1002/cld.857 PMC6924966

[4] Moreau R Jalan R Gines P , et al. Acute-on-chronic liver failure is a distinct syndrome that develops in patients with acute decompensation of cirrhosis. Gastroenterology 2013; 144(7): 1426–1437, 1437.e1–9. 10.1053/j.gastro.2013.02.042 23474284

[5] Jalan R D’Amico G Trebicka J , et al. New clinical and pathophysiological perspectives defining the trajectory of cirrhosis. J Hepatol 2021; 75(Suppl. 1): S14–S26. 10.1016/j.jhep.2021.01.018 34039485

[6] Trebicka J Fernandez J Papp M , et al. The PREDICT study uncovers three clinical courses of acutely decompensated cirrhosis that have distinct pathophysiology. J Hepatol 2020; 73(4): 842–854. 32673741 10.1016/j.jhep.2020.06.013

[7] Mokdad AA Lopez AD Shahraz S , et al. Liver cirrhosis mortality in 187 countries between 1980 and 2010: a systematic analysis. BMC Med 2014; 12: 145. 25242656 10.1186/s12916-014-0145-y PMC4169640

[8] GBD 2017 Cirrhosis Collaborators. The global, regional, and national burden of cirrhosis by cause in 195 countries and territories, 1990–2017: a systematic analysis for the Global Burden of Disease Study 2017. Lancet Gastroenterol Hepatol 2020; 5(3): 245–266. 31981519 10.1016/S2468-1253(19)30349-8 PMC7026710

[9] Tapper EB Parikh ND . Mortality due to cirrhosis and liver cancer in the United States, 1999–2016: observational study. BMJ 2018; 362: k2817. 10.1136/bmj.k2817 PMC6050518 30021785

[10] Roth K Lynn J Zhong Z , et al. Dying with end stage liver disease with cirrhosis: insights from SUPPORT. Study to understand prognoses and preferences for outcomes and risks of treatment. J Am Geriatr Soc 2000; 48(S1): S122–S130. 10809465

[11] Peng J-K Hepgul N Higginson IJ , et al. Symptom prevalence and quality of life of patients with end-stage liver disease: a systematic review and meta-analysis. Palliat Med 2019; 33(1): 24–36. 30345878 10.1177/0269216318807051 PMC6291907

[12] Radbruch L De Lima L Knaul F , et al. Redefining palliative care—a new consensus-based definition. J Pain Symptom Manage 2020; 60(4): 754–764. 32387576 10.1016/j.jpainsymman.2020.04.027 PMC8096724

[13] Sepúlveda C Marlin A Yoshida T , et al. Palliative care: the World Health Organization’s global perspective. J Pain Symptom Manage 2002; 24(2): 91–96. 12231124 10.1016/s0885-3924(02)00440-2

[14] Baumann AJ Wheeler DS James M , et al. Benefit of early palliative care intervention in end-stage liver disease patients awaiting liver transplantation. J Pain Symptom Manage 2015; 50(6): 882–886.e2. 10.1016/j.jpainsymman.2015.07.014 26303186

[15] Verma M Tapper EB Singal AG , et al. Nonhospice palliative care within the treatment of end-stage liver disease. Hepatology 2020; 71(6): 2149–2159. 32167615 10.1002/hep.31226 PMC10362480

[16] Shehadah A Yu Naing L Bapaye J , et al. Early palliative care referral may improve end-of-life care in end-stage liver disease patients: a retrospective analysis from a non-transplant center. Am J Med Sci 2024; 367(1): 35–40. 37923293 10.1016/j.amjms.2023.10.006

[17] Rogal SS Hansen L Patel A , et al. AASLD Practice Guidance: palliative care and symptom-based management in decompensated cirrhosis. Hepatology 2022; 76(3): 819–853. 35103995 10.1002/hep.32378 PMC9942270

[18] Singal AG Llovet JM Yarchoan M , et al. AASLD Practice Guidance on prevention, diagnosis, and treatment of hepatocellular carcinoma. Hepatology 2023; 78(6): 1922–1965. 37199193 10.1097/HEP.0000000000000466 PMC10663390

[19] Ufere NN Donlan J Waldman L , et al. Barriers to use of palliative care and advance care planning discussions for patients with end-stage liver disease. Clin Gastroenterol Hepatol 2019; 17(12): 2592–2599. 30885884 10.1016/j.cgh.2019.03.022 PMC6745282

[20] Beck KR Pantilat SZ O’Riordan DL , et al. Use of palliative care consultation for patients with end-stage liver disease: survey of liver transplant service providers. J Palliat Med 2016; 19(8): 836–841. 27092870 10.1089/jpm.2016.0002 PMC5335746

[21] Gómez-Batiste X Martínez-Muñoz M Blay C , et al. NECPAL CCOMS-ICO^©^ tool version 1.0. Tool to identify advanced-terminal patients in need of palliative care within health and social services, https://ico.gencat.cat/web/.content/minisite/ico/professionals/documents/qualy/arxius/necpal_tool_eng_vf.pdf (2011, accessed 9 December 2024).

[22] The SPICT™ Programme. Supportive & palliative care indicators tool (SPICT), https://www.spict.org.uk/the-spict/ (2022, accessed 11 December 2024).

[23] Tandon P Reddy KR O’Leary JG , et al. A Karnofsky performance status-based score predicts death after hospital discharge in patients with cirrhosis. Hepatology 2017; 65(1): 217–224. 27775842 10.1002/hep.28900

[24] Hudson BE Ameneshoa K Gopfert A , et al. Integration of palliative and supportive care in the management of advanced liver disease: development and evaluation of a prognostic screening tool and supportive care intervention. Frontline Gastroenterol 2017; 8(1): 45–52. 28839884 10.1136/flgastro-2016-100734 PMC5369436

[25] Brown C Aksan N Muir AJ . Consider hospice in end-stage liver disease prognostic scale to open discussions regarding six-month mortality. JGH Open 2023; 7(4): 278–285. 37125249 10.1002/jgh3.12889 PMC10134759

[26] Kamath PS Wiesner RH Malinchoc M , et al. A model to predict survival in patients with end-stage liver disease. Hepatology 2001; 33(2): 464–470. 11172350 10.1053/jhep.2001.22172

[27] Forner A Reig M Bruix J . Hepatocellular carcinoma. Lancet 2018; 391(10127): 1301–1314. 29307467 10.1016/S0140-6736(18)30010-2

[28] von Elm E Altman DG Egger M , et al. Strengthening the Reporting of Observational Studies in Epidemiology (STROBE) statement: guidelines for reporting observational studies. BMJ 2007; 335(7624): 806–808. 17947786 10.1136/bmj.39335.541782.AD PMC2034723

[29] Charlson ME Pompei P Ales KL , et al. A new method of classifying prognostic comorbidity in longitudinal studies: development and validation. J Chronic Dis 1987; 40(5): 373–383. 3558716 10.1016/0021-9681(87)90171-8

[30] Pugh RN Murray-Lyon IM Dawson JL , et al. Transection of the oesophagus for bleeding oesophageal varices. Br J Surg 1973; 60(8): 646–649. 4541913 10.1002/bjs.1800600817

[31] Crooks V Waller S Smith T , et al. The use of the Karnofsky Performance Scale in determining outcomes and risk in geriatric outpatients. J Gerontol 1991; 46(4): M139–M144. 10.1093/geronj/46.4.m139 2071835

[32] Bruera E Kuehn N Miller MJ , et al. The Edmonton Symptom Assessment System (ESAS): a simple method for the assessment of palliative care patients. J Palliat Care 1991; 7(2): 6–9. 1714502

[33] Hui D Bruera E . The Edmonton symptom assessment system 25 years later: past, present, and future developments. J Pain Symptom Manage 2017; 53(3): 630–643. 28042071 10.1016/j.jpainsymman.2016.10.370 PMC5337174

[34] Donlan J Zeng C Indriolo T , et al. The Edmonton symptom assessment system is a valid, reliable, and responsive tool to assess symptom burden in decompensated cirrhosis. Hepatol Commun 2024; 8(4): e0385. 10.1097/HC9.0000000000000385 PMC10948137 38497942

[35] Chen H Johnston A Palmer A , et al. Too little, too late: palliation and end-stage liver disease. J Gastroenterol Hepatol 2021; 36(8): 2303–2306. 33738858 10.1111/jgh.15499

[36] Medici V Rossaro L Wegelin JA , et al. The utility of the model for end-stage liver disease score: a reliable guide for liver transplant candidacy and, for select patients, simultaneous hospice referral. Liver Transpl 2008; 14(8): 1100–1106. 18668666 10.1002/lt.21398

[37] Barnes A Woodman RJ Kleinig P , et al. Early palliative care referral in patients with end stage liver disease is associated with reduced resource utilisation. J Gastroenterol Hepatol. Epub ahead of print October 2019. DOI: 10.1111/jgh.14877. 31613397

[38] Kathpalia P Smith A Lai JC . Underutilization of palliative care services in the liver transplant population. World J Transplant 2016; 6(3): 594–598. 27683638 10.5500/wjt.v6.i3.594 PMC5036129

[39] Hansen L Chang MF Hiatt S , et al. Symptom classes in decompensated liver disease. Clin Gastroenterol Hepatol 2022; 20(11): 2551–2557.e1. 10.1016/j.cgh.2021.11.023 PMC9120261 34813941

[40] Rush B Fruhstofer C Walley KR , et al. Palliative medicine and hospital readmissions in end-stage liver disease. BMJ Support Palliat Care. Epub ahead of print February 2019. DOI: 10.1136/bmjspcare-2018-001635. 30760459

[41] English MR Ellis J Verma S , et al. Outcomes in cirrhosis-related refractory ascites with emphasis on palliative care: single-centre experience and literature review. Curr Hepatol Rep 2024; 23: 316–324. 41194902 10.1007/s11901-024-00669-0 PMC7618328

[42] O’Leary JG Tandon P Reddy KR , et al. Underutilization of hospice in inpatients with cirrhosis: the NACSELD experience. Dig Dis Sci 2020; 65(9): 2571–2579. 32146602 10.1007/s10620-020-06168-8

[43] Woodland H Buchanan RM Pring A , et al. Inequity in end-of-life care for patients with chronic liver disease in England. Liver Int 2023; 43(11): 2393–2403. 37519025 10.1111/liv.15684

[44] Deng LX Bischoff KE Kent DS , et al. Frailty is strongly associated with self-reported symptom burden among patients with cirrhosis. Eur J Gastroenterol Hepatol 2021; 33(1S Suppl. 1): e395–e400. 10.1097/MEG.0000000000002113 PMC8435043 33731588

[45] Tandon P Tangri N Thomas L , et al. A rapid bedside screen to predict unplanned hospitalization and death in outpatients with cirrhosis: a prospective evaluation of the Clinical Frailty Scale. Am J Gastroenterol 2016; 111(12): 1759–1767. 27481305 10.1038/ajg.2016.303

[46] Tonon M D’Ambrosio R Calvino V , et al. A new clinical and prognostic characterization of the patterns of decompensation of cirrhosis. J Hepatol 2024; 80(4): 603–609. 38110003 10.1016/j.jhep.2023.12.005

[47] Adejumo AC Kim D Iqbal U , et al. Suboptimal use of inpatient palliative care consultation may lead to higher readmissions and costs in end-stage liver disease. J Palliat Med 2020; 23(1): 97–106. 31397615 10.1089/jpm.2019.0100 PMC6931914

[48] Sohal A Chaudhry H Sharma R , et al. Recent trends in palliative care utilization in patients with decompensated liver disease: 2016–2020 national analysis. J Palliat Med 2024; 27(3): 335–344. 37851991 10.1089/jpm.2023.0367

[49] Holden JH Shamseddeen H Johnson AW , et al. Palliative care and hospice referrals in patients with decompensated cirrhosis: what factors are important? J Palliat Med 2020; 23(8): 1066–1075. 32091954 10.1089/jpm.2019.0501 PMC7404816

[50] Patel AA Woodrell C Ufere NN , et al. Developing priorities for palliative care research in advanced liver disease: a multidisciplinary approach. Hepatol Commun 2021; 5(9): 1469–1480. 34510839 10.1002/hep4.1743 PMC8435283

[51] Malik S Arutla V Alamin T , et al. Beyond the diagnosis: a deep dive into the end stage liver disease experience from the patient perspective. Am J Hosp Palliat Care 2025; 42(4): 389–395. 38780457 10.1177/10499091241256629

[52] Kieffer SF Tanaka T Ogilvie AC , et al. Palliative care and end-of-life outcomes in patients considered for liver transplantation: a single-center experience in the US Midwest. Am J Hosp Palliat Care 2023; 40(10): 1049–1057. 36448659 10.1177/10499091221142841

[53] Oliveira HM Miranda HP Rego F , et al. Palliative care and end stage liver disease: a cohort analysis of palliative care use and factors associated with referral. Ann Hepatol 2024; 29(5): 101518. 38851396 10.1016/j.aohep.2024.101518

[54] Carvalho JR Vasconcelos M Marques da Costa P , et al. Identifying palliative care needs in a Portuguese liver unit. Liver Int 2018; 38(11): 1982–1987. 29682885 10.1111/liv.13865

[55] Shinall MC Jr Karlekar M Martin S , et al. COMPASS: a pilot trial of an early palliative care intervention for patients with end-stage liver disease. J Pain Symptom Manage 2019; 58(4): 614–622.e3. 10.1016/j.jpainsymman.2019.06.023 PMC6754773 31276810

